# The mechanism of action of *Ophiocordyceps sinensis* mycelia for prevention of acute lung injury based on non-targeted serum metabolomics

**DOI:** 10.1371/journal.pone.0287331

**Published:** 2023-06-16

**Authors:** Jinna Zhou, Na Pi, Yingqi Guo, Xinyi He, Jinhu Wang, Run Luo, Mu Wang, Hong Yu

**Affiliations:** 1 College of Ecology and Environment Sciences, Yunnan University, Kunming, China; 2 School of Ecology and Environment, Tibet University, Lhasa City, China; 3 Plant Science College, Tibet Agriculture & Animal Husbandry University, Nyingchi, China; 4 Institute of Medical Biology, Chinese Academy of Medical Sciences, Kunming, China; 5 Institutional Center for Shared Technologies and Facilities of Kunming Institute of Zoology, Chinese Academy of Sciences, Kunming, China; Helwan University, EGYPT

## Abstract

*Ophiocordyceps sinensis* is a fungus with medicinal value in treating lung diseases, but no study has reported how to prevent acute lung injury using this fungus. The mice were divided into normal, model, positive control, and *O*. *sinensis* groups to observe lung histopathological sections and transmission electron microscopy, along with liquid chromatography-mass spectrometry and hematoxylin and eosin (H&E) staining to closely identify structural differences resulting from destruction between the groups. The results of the H&E staining showed that, compared with the normal group, the model group showed alveolar collapse. Compared with the model group, the infiltration of inflammatory cells in the alveolar cavity of the *O*. *sinensis* group was significantly reduced. Mitochondrial plate-like cristae were observed in type II alveolar cells of the normal group, with normal coloration of the mitochondrial matrix. Type II alveolar cells in the model group showed obvious edema. The statuses of type II alveolar cells in the *O*. *sinensis* and positive groups were similar to that in the normal group. Twenty-nine biomarkers and 10 related metabolic pathways were identified by serum metabolomics screening. The results showed that *O*. *sinensis* mycelia had a significant effect on the prevention of lipopolysaccharide-induced inflammation.

## 1. Introduction

Acute lung injury (ALI) is one of the most common acute and critical clinical conditions caused by gram-negative bacterial infection, bacterial outer membrane lipopolysaccharide activation of inflammatory cells, release of a large number of inflammatory factors, and further development of acute respiratory distress syndrome (ARDS) [1, 2]. The pathogenesis of ALI is often accompanied by pulmonary edema and accumulation of inflammatory cells, and the pathogenesis of novel coronavirus pneumonia (COVID-19) is closely related to ALI development. Currently, no specific drug is available for this disease in clinical practice [3]. Therefore, the discovery of herbs with the potential to prevent ALI is crucial. Lipopolysaccharide (LPS) is the main toxic component of the cell wall of gram-negative bacteria. When the body suffers from a severe infection or multifactorial injury that increases the permeability of the intestinal mucosa, sepsis can develop due to an increase in LPS [4], which can further induce ALI. The current animal models for the induction of ALI mostly involve the intraperitoneal injection of LPS, but intraperitoneal injection does not adequately simulate the pathogenesis and mechanism of ALI. Therefore, this experiment used tracheal drip LPS to induce an in vivo model of ALI.

Chinese cordyceps is a fungal complex formed by parasitic *O*. *sinensis* mushrooms on the larvae of insects of the bat moth family, mainly distributed on the Tibetan plateau 2500 m above sea level. Currently, *O*. *sinensis* mycelia are cultivated in vitro and is widely used as a substitute for *O*. *sinensis* in pharmaceutical and herbal products [5]. *O*. *sinensis* has a long history in the treatment of lung diseases [6–8], and modern pharmacology confirms that it has a very positive effect on chronic obstructive pulmonary disease (COPD), asthma, and pulmonary interstitial fibrosis (PIF) [9]. However, studies on the prevention of LPS-induced ALI using *O*. *sinensis* are lacking. Therefore, in this experiment tracheal drip LPS was used to induce an in vivo model of ALI, which could contribute to the prevention of COVID-19.

Metabolomics is a major branch of histology [10, 11] and is mainly used to study dynamic changes in various small molecule metabolites, including amino acids and fatty acids. High-throughput chromatography and mass spectrometry techniques, combined with statistical methods for data analysis, are used to identify and quantify metabolites in biological samples, thus reflecting the overall functions of complex living organisms. The metabolic profile of disease occurrence and drug treatment can be clearly obtained from the holistic, correlative, and dynamic nature of metabolomics in the analysis. In recent years, metabolomics has been increasingly used in the field of medicine [12–14], particularly in the study of disease mechanisms [15, 16]. Combined with metabolomics, studying the prevention of ALI by *O*. *sinensis* mycelia is of great significance.

## 2. Materials and methods

### 2.1 Preparation of *O*. *sinensis* mycelium solution

We added 100 g of potato powder, 10 g of glucose, 9 g of agar, 5 g of yeast, and 2.5 g of peptone to 500 mL of distilled water. The mixture was sterilized at 121°C at 10^5^ Pa for 30 min and introduced into a culture dish in a sterile environment to form 24 solid media. Wild *O*. *sinensis* collected from the Baima Snow Mountain in Yunnan Province in June 2022 was inoculated onto the culture medium using tissue separation method. A room at 16.5°C for 4 months to obtain *O*. *sinensis* mycelium was incubated. After further removing the culture medium, 20 g of fresh *O*. *sinensis* mycelia was obtained by artificial culture, dried at 45°C for 10 h, ground into fine powder, and passed through a 100-mesh sieve. Distilled water was added to 1.5 g of *O*. *sinensis* mycelia to make 45 mg/mL of *O*. *sinensis* mycelium solution. The solution was set aside at 4°C [17].

### 2.2 Grouping of animals and drug administration

The animals were housed at a temperature of 20±2°C and relative humidity of 55±5%, with a 12-h alternating day and night environment and free access to food and water. Animal experiments were approved by the Medical Ethics Committee of Yunnan University (approval number: YNU20230536). In total, 24 mice were randomly divided into the normal (NS), model (LPS), *O*. *sinensis* mycelia (OS) groups, and dexamethasone solution was used for positive control (DXM) groups. On the first day of the experiment, mice in the NS and LPS groups were intraperitoneally injected with physiological saline, and the OS and DXM groups were injected with *O*. *sinensis* mycelium solution (45 mg/mL) and dexamethasone solution, respectively, for 7 consecutive days. On the 7^th^ day, mice in the LPS, OS, and DXM groups were anesthetized by an intraperitoneal injection of pentobarbital sodium for 1 h. The trachea of mice was exposed through surgery, and tracheal puncture was performed using a miniature sampler. LPS solution (5 mg/kg) was dropped into the body of mice to establish an acute lung injury model. In the NS group, after administering physiological saline for 1 h on the 7^th^ day, the mice were anesthetized with an intraperitoneal injection of pentobarbital sodium for 1 h, and sterile saline was dripped with a mini-syringe for tracheal puncture. Blood was collected from the orbital plexus of each of the mice 6 h after the establishment of an acute lung injury model in each group, the collected blood was placed in ice cubes, and the serum was separated after centrifugation at 3500 r*min-1 for 10 min and stored in a refrigerator at −80°C.

### 2.3 LPS-induced ALI

In the experimental LPS-induced ALI, mice were first anesthetized by an intraperitoneal injection of 1% sodium pentobarbital (30 mg/kg). The limbs and teeth of the anesthetized mice were fixed and disinfected by wiping with 75% alcohol, and an incision was made longitudinally along the central neck of the mice to expose the tracheal cartilage ring. The microinjector was fed toward the proximal level, LPS was slowly injected, and sterile saline was injected into the NS group. The microsampler was withdrawn, and the mice were immediately placed upright and rotated for 2 weeks before suturing the wound.

### 2.4 Lung wet/dry weight ratio

The body masses of the mice were weighed before sampling. The mice were deeply anesthetized, and the entire lung was taken out. Residual blood was aspirated using wet cotton. The weight of the middle lobe of the right lung was measured and recorded (marked as wet weight). Afterwards, the sample was placed in an oven at a temperature of 60°C for 24 consecutive h. The weight was measured and recorded again (marked as dry weight), and the lung coefficient was calculated according to the following formula: lung coefficient/% = lung wet/dry weight ratio × 100%.The results were analyzed statistically using SPSS 22.0 software (IBM Corp.; Armonk, NY, USA), and the data obeying the measures were expressed as x ± sd. If the squares were equal, a one-way analysis of variance and q-test were performed; if the squares were not equal, the analysis was performed by the rank sum test for multiple sample comparison, and two independent samples were analyzed by the rank sum test for two-group comparison.

### 2.5 H&E staining for lung histopathological changes

The lung samples were fixed in 10% formalin and embedded in paraffin. Tissue blocks were cut into 5-μm slices, stained with hematoxylin and eosin (H&E), and analyzed under a light microscope.

### 2.6 Observation and analysis under a light microscope

The samples were fixed overnight at 4°C using 2.5% glutaraldehyde in 0.1-M PB (PH, 7.2) and washed with 0.1-M PB (PH, 7.2) three times for 7 min. Afterwards, samples were postfixed with 1% OsO_4_ and 1.5% K_4_Fe(CN)_6_ for 2 h at 4°C and washed with ddH_2_O three times for 7 min, followed by serial ethanol dehydration and acetone transition for 5 min. They were embedded in SPI pon 812 resin and underwent polymerization at 60°C for 48 h. Serial sections with uniform thicknesses (60 nm) were prepared using a Leica EM UC7 ultramicrotome. Ultrathin sections were loaded onto copper grids and double-stained with 2% uranyl acetate and lead citrate before observation under a JEM-1400 Plus transmission electron microscope at 80 kv.

### 2.7 Serum metabolite extraction

During the sample mass spectrometry process, 9 quality control (QC) samples were interspersed. Data quality was evaluated through the repeatability of QC sample testing. Serum samples, stored at −80°C, including sample preparation QC, were placed in a refrigerator at 4°C to thaw until no ice cubes were visible. Thereafter, 100 μL of each sample (including QC) were added to the EP tube, and the remaining samples were frozen. Subsequently, 700 μL of extractant containing internal standard 1 (methanol: acetonitrile: water = 4:2:1, V/V/V) was added, shaken for 1 min, and placed in a refrigerator at −20°C for 2 h. The samples were centrifuged at 25,000 × g at 4°C for 15 min. Samples were subsequently removed from the centrifuge, and 600 μL of the supernatant was transferred to a split new EP tube. A drying machine was used for drying, and 180 μL of methanol: pure water (1:1 v/v) solution was added and vortexed for 10 min until all dissolved in the reconstituted solution. It was subsequently centrifuged at 25,000 × g at 4°C for 15 min. The supernatant was moved to a new EP tube, and 20 μL of each sample was mixed into QC samples. The prepared supernatant was subjected to LC-MS/MS.

### 2.8 UPLC-MS analysis

A Water 2777c UPLC (Waters, USA) in series with a Q Exactive HF high-resolution mass spectrometer (Thermo Fisher Scientific) was used for the separation and detection of metabolites. Chromatographic conditions were as follows: Chromatographic separation was performed on a Waters ACQUITY UPLC BEH C18 column (1.7 μm, 2.1 mm × 100 mm, Waters, USA), and the column temperature was maintained at 45°C [18]. The mobile phase comprised 0.1% formic acid (A) and acetonitrile (B) in the positive mode and 10 mM ammonium formate (A) and acetonitrile (B) in the negative mode. The gradient conditions were as follows: 0–1 min, 2% B; 1–9 min, 2%–98% B; 9–12 min, 98% B; 12–12.1 min, 98%–2% B; and 12.1–15 min, 2% B. The flow rate was 0.35 mL/min, and the injection volume was 5 μL. Mass spectrometry conditions were as follows: Primary and secondary mass spectrometry data were acquired using Q Exactive HF (Thermo Fisher Scientific, USA). The full scan range was 70–1050 m/z with a resolution of 120,000, and the automatic gain control (AGC) target for MS acquisition was set to 3e6 with a maximum ion injection time of 100 ms. The top three precursors were selected for subsequent MSMS fragmentation with a maximum ion injection time of 50 ms and a resolution of 30,000; the AGC was 1e5. The stepped normalized collision energies were set as 20, 40, and 60 eV. ESI parameters were set as follows: the sheath gas flow rate was 40; auxiliary gas flow rate was 10; positive-ion mode, spray voltage (|KV|) was 3.80; negative-ion mode, spray voltage (|KV|) was 3.20; capillary temperature was 320°C; and auxiliary gas heater temperature was 350°C [19].

### 2.9 Metabolite ion peak extraction and metabolite identification

After importing the offline mass spectrometry data into Compound Discoverer v.3.3 (Thermo Fisher Scientific, USA) and analyzing the mass spectrometry data, in combination with the BGI metabolome database (bmdb), mzcloud database, and chemspider online database, a data matrix containing information such as metabolite peak area and identification results was obtained. Subsequently, the table was further analyzed and processed. Parameters were set as follows: parent ion mass deviation, <5 ppm; mass deviation of fragment ions, <10 ppm; and retention time deviation, <0.2 min. The official website link is https://mycompounddiscoverer.com/. The statistics of the metabolites with identification information were based on their final classes.

### 2.10 Data preprocessing and statistical analysis

The result file from Compound Discoverer was input into MetaX for data preprocessing and further analysis. Data preprocessing included the following steps: 1) Data was normalized to obtain relative peak areas by probabilistic quotient normalization [20]. 2) Using quality control-based robust LOESS [9], signal correction was implemented to correct for batch effects. 3) Metabolites were removed with a coefficient of variation >30% of their relative peak areas in the removed QC samples. PCA and PLS-DA were performed using SIMCA software Version 14.1 (Umetrics AB, Umeå, Sweden). The fold change of each metabolite in each comparison group was calculated, and Student’s t-test was used to test the significance of the expression of each metabolite in each comparison group to obtain p-values. A *P*-value was used to evaluate the significance level of the difference between the two groups of samples. Fold change measures whether the mean value of metabolites in both sample groups changed, and the p-value measures whether the change is statistically significant. Only metabolites with fold change values of ≥1.2 or ≤0.83 and *P*-values of <0.05 can be classified as differential metabolites. The metabolic pathway analysis of the differentially expressed metabolites was performed by the online tool MetaboAnalyst (https://www.metaboanalyst.ca/ [accessed on 3 March 2023]).

## 3. Results

### 3.1 Lung wet/dry weight ratio

**Fig 1:**
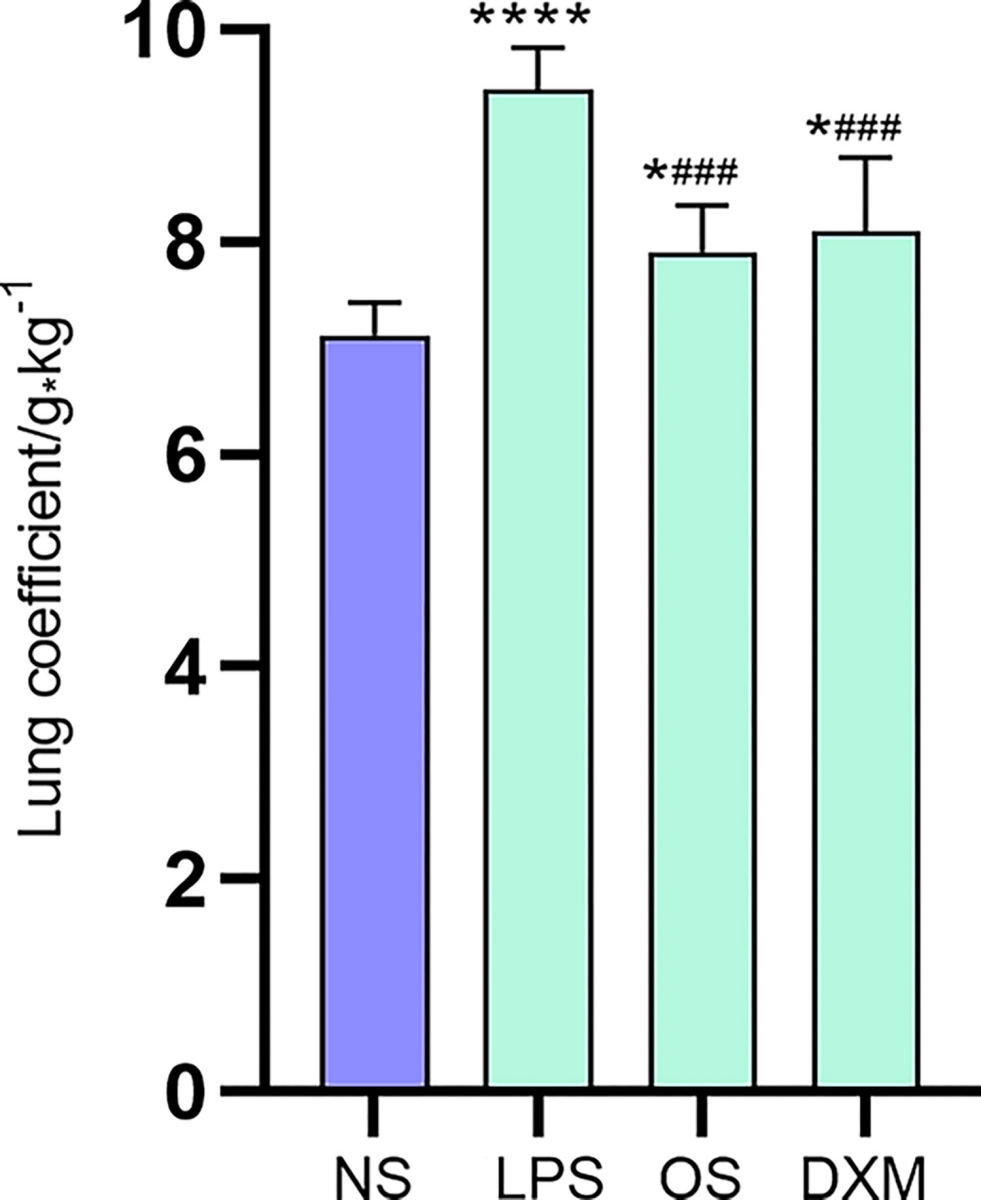
Presents the lung coefficient (n = 5). LPS group vs. NS group, **** *P* <0.001; LPS group vs. OS group, * *P* <0.01, LPS group vs. DXM group, * *P* <0.01; NS group vs. OS group, ^###^ *P* <0.05, NS group vs. DXM group, ^###^ *P* <0.05.

The results of lung coefficients showed that the lung coefficients of mice in the LPS group were significantly higher than those in the NS group (*P* <0.001, Fig 1). Compared with the LPS group, the lung index of the OS and DXM groups showed a decreasing trend (*P*<0.01), while the lung index of the OS and DXM groups also showed an increase compared with the NS group *(P* <0.05). It was concluded that *O*. *sinensis* mycelia could reduce the lung coefficients in mice, and its effect was comparable to that of DXM.

### 3.2 H&E stain

**Fig 2:**
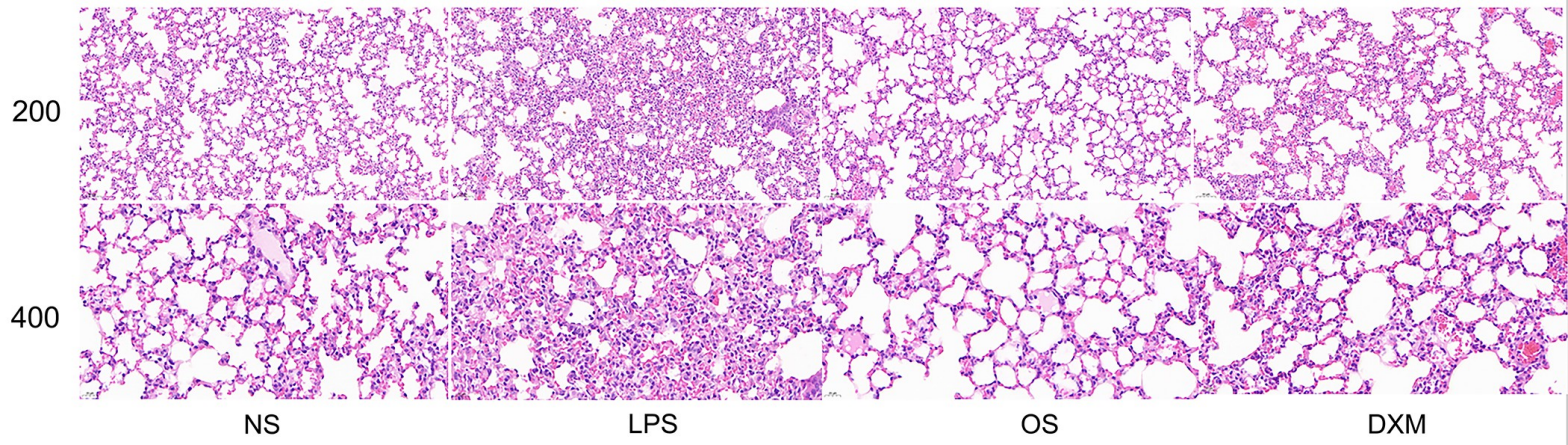
Representative H&E-stained lung sections (n = 5), scar bar = 20 μm.

The H&E staining results showed that the lung tissue structure of the NS group mice was clear, with no inflammatory cell exudation in the alveolar cavity and no thickening of the alveolar septa. Compared with the NS group, the model group showed alveolar collapse and thickening of alveolar walls and was accompanied by alveolar edema and significant inflammatory cell infiltration. Compared with the LPS group, the OS group had fewer alveolar collapses, no thickening of alveolar walls, and no significant infiltration of inflammatory cells, and the corresponding efficacy was comparable to that of DXM (Fig 2).

### 3.3 Transmission electron microscopy

**Fig 3:**
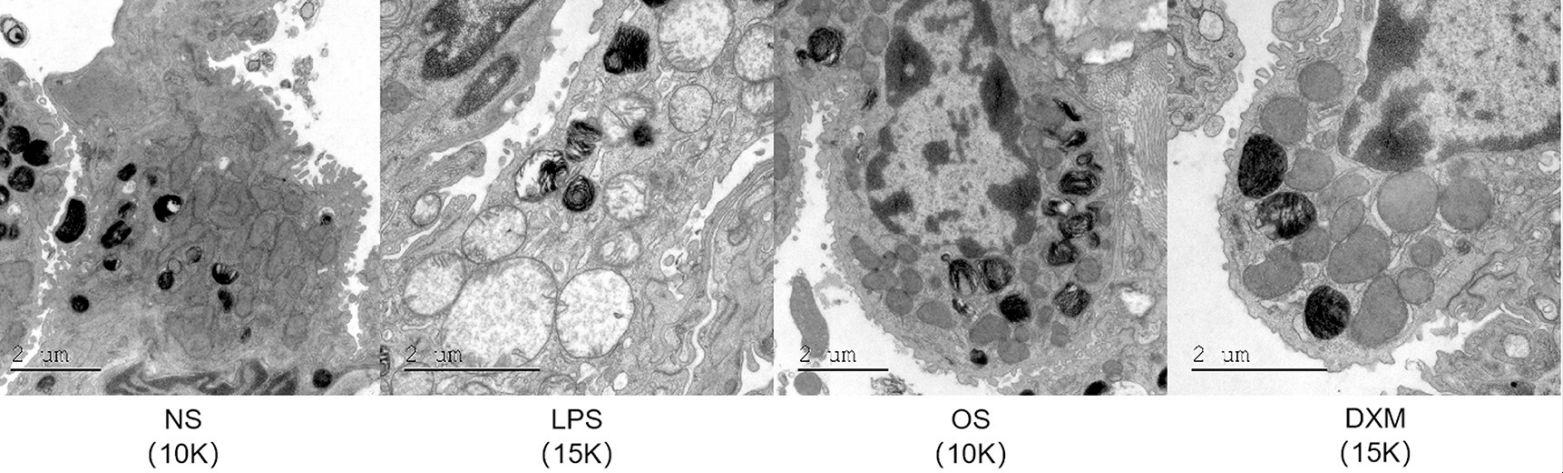
Ultrastructural changes in the lung tissue of mice in each group. LB, platelet vesicles; M, mitochondria; and↑ endoplasmic reticulum.

The transmission electron microscopy results were consistent with the HE staining results (Fig 3). At the subcellular level, we focused on type II alveolar cells, which contain layered secretory granules containing phospholipids, proteins, and glycosaminoglycans. It has the function of synthesizing and secreting pulmonary surfactants, as well as maintaining the alveolar environment. In type II alveolar cells of the NS group, mitochondrial lamellar cristae were visible, mitochondrial matrix was stained normally, rough endoplasmic reticulum was flat and cystic, ribosome was attached to the surface, lamellar bodies were in normal shape, and microvilli were abundant on the cell surface. In the LPS group, the type II alveolar cells showed obvious edema, which was manifested as the expansion of the inner chamber of mitochondria, the fracture of the crista, and the obvious decrease of electron density, presenting a vacuolar shape. Simultaneously, the lamellar bodies became vacuolated, and microvilli atrophy decreased. The status of type II alveolar cells in the OS group and DXM group was similar to that in NS group, but the microvilli were atrophied and decreased. There was still slight edema in mitochondria and endoplasmic reticulum in the DXM group. The experimental results indicate that the mycelium of *O*. *sinensis* mycelia has a significant preventive effect on LPS-induced inflammation.

### 3.4 Serum metabolome

#### 3.4.1 Total ion flow diagram for the LC-MS analysis of mouse serum

**Fig 4:**
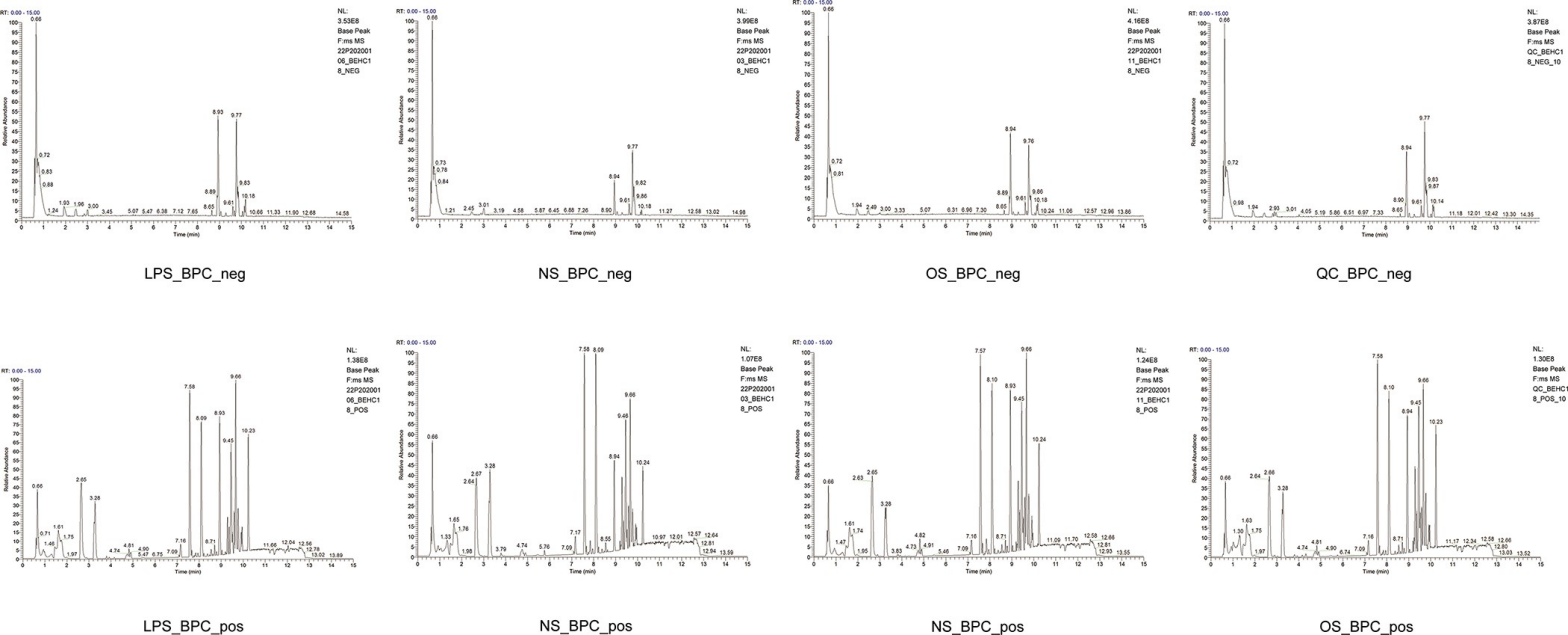
(**a**) Typical peak chromatograms of each group of samples. (**b**) Metabolite classification bar chart.

A typical base peak chromatogram (BPC) for each group of samples in positive and negative ion modes provided a visual representation of metabolite detection in the sample (Fig 4A). Usually, more ion peaks in the BPC graph indicated more metabolites. The total ion flow chromatogram obtained from the serum samples is shown in the figure. The ion intensity of the highest peak in the graph was 100%, and the separation between the spectral peaks in the graph was good. From the LC-MS spectrum, 1,153 metabolites were identified, including phytochemical compounds, compounds with biological roles, lipids, and others. The plant compounds included terpenoids, flavonoids, and alkaloid compounds with biological roles, including amino acids, peptides, analogs, benzene and its derivatives, organic acids, and steroids and its derivatives (Fig 4B).

#### 3.4.2 PCA

**Fig 5:**
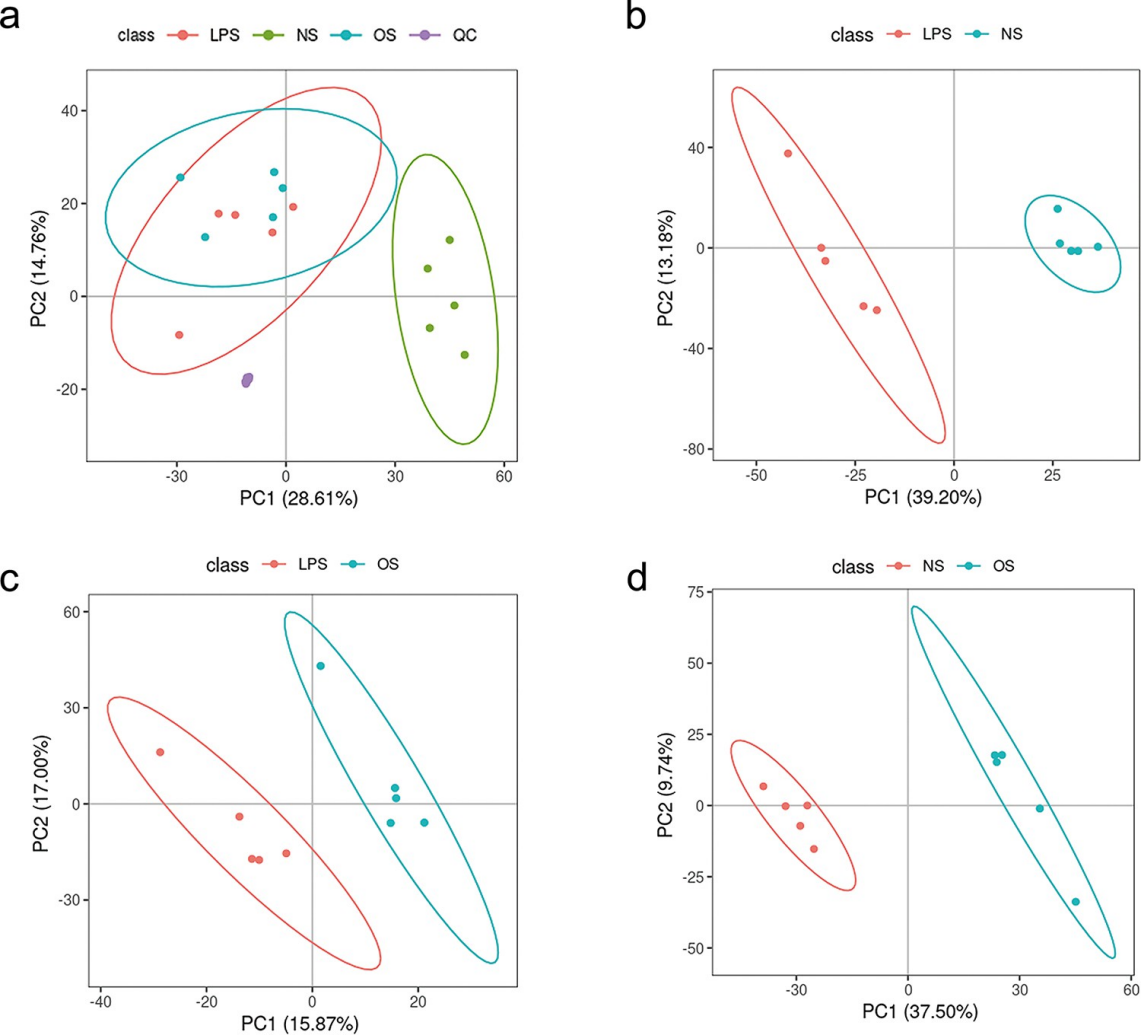
**(a)** Score chart of the PCA analysis model; **(b)** score chart of the PLS-DA analysis model of LPS_NS; **(c)** score chart of the PLS-DA analysis model of LPS_OS; and **(d)** score chart of the PLS-DA analysis model of NS_OS.

The samples in each group were tightly clustered and concentrated in positive and negative ion modes. The results indicated that the precision and reproducibility of this experiment and stability of the assay system were good. Serum samples were well clustered within each of the NS, LPS, and OS groups, and a trend of separation of serum samples in each group was identified (Fig 5A), suggesting that the serum metabolic profiles of mice fed with *O*. *sinensis* mycelia changed. The better clustering of QC samples indicates that the more stable the instrument, the better the reproducibility of the collected data.

#### 3.4.3 PLS-DA

PLS-DA analysis was performed on the two groups of biological samples to model the relationship between metabolite expression and sample class, which allowed the prediction of the sample class. The model parameters R^2^Y = 1 and Q^2^ = 0.92 for the LPS and NS control groups (Fig 5B), R^2^Y = 0.99 and Q^2^ = 0.52 for the LPS and OS control groups (Fig 5C), and R^2^Y = 0.1 and Q^2^ = 0.9 for the LPS and NS control groups are valid and reliable (Fig 5D).

#### 3.4.4 Differential metabolite screening

**Fig 6:**
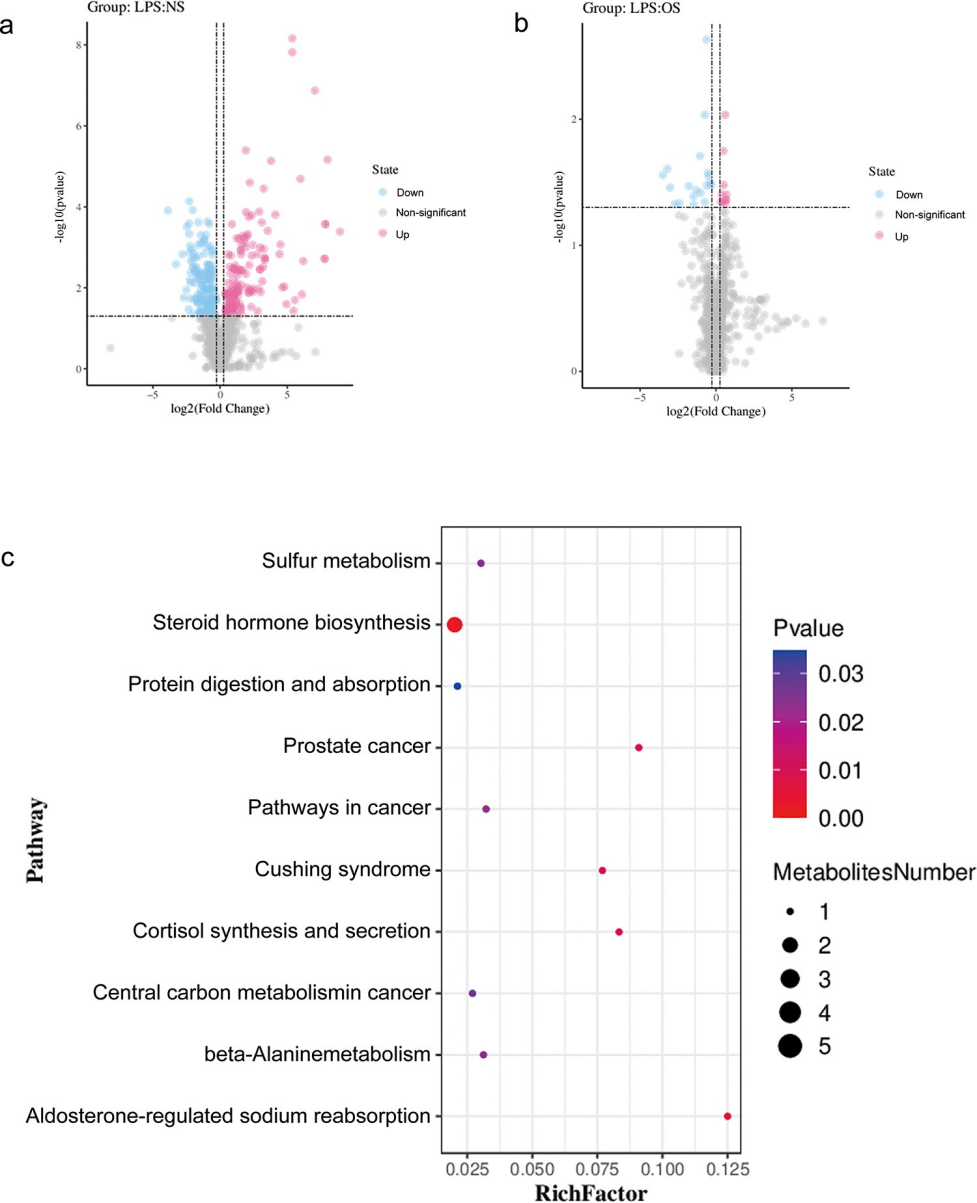
**(a)** Volcano map of differential metabolites of LPS_NS; **(b)** volcano map of differential metabolites of LPS_OS; and **(c)** bubble diagram for metabolic pathway enrichment analysis.

The results indicated the presence of 304 differential metabolites in the LPS group compared with those in the NS group, of which 149 were upregulated, including cytosine (Fig 6A), 3-hydroxyanthranilic acid, crotonic acid, thymidine, 3-methoxybenzaldehyde, hexanoylglycine, n-acetyl-d-galactosamine, sebacate, 4-methoxycinnamaldehyde, capryloylglycine, cortisol, 3-hydroxydecanoic acid, corticosterone, and tetrahydrocortisone. In total, 155 metabolites were down-regulated, including l-lysine, (r)-malate, succinate, l-methionine, l-tryptophan, adipate, 4-hydroxy-2-l-thyroxine, and gamma-linolenic acid, suggesting that the 304 metabolites are associated with LPS-induced lung injury in mice. Further, 29 differential metabolites were found in the LPS and OS groups (Fig 6B), including l-histidine, n-isovalerylglycine, indole-3-ethanol, dl-histidine, (hydroxyethyl)methacrylate, n-acetylvaline, n(6)-[(indol-3-yl)acetyl]-l-lysine, rac-etomidate, and 10 other metabolites that showed upregulation, and cortisol, tetrahydrocortisone, apocynin, 3-hydroxy-3-methylglutaric acid, dimethyl sulfoxide, piperonyl sulfoxide, niaprazine, 1-stearoyl-2 arachidonoylplasmenylcholine, gibberellin a24, hydrocortisone cypionate, butylparaben, 7-ketodeoxycholic acid, (-)-nabilone, a12(13)-epode, and 19 other metabolites that showed down-regulation. It has been suggested that *O*. *sinensis* mycelia exerts preventive effects by regulating the in vivo levels of these metabolites.

#### 3.4.5 Metabolic pathway analysis

Enrichment of the 29 screened *O*. *sinensis* mycelia biomarkers for the prevention of acute lung injury in mice showed that these metabolites were mainly enriched in ten metabolic pathways (Fig 6C), including the steroid hormone biosynthesis, aldosterone-regulated sodium reabsorption in prostate cancer, cortisol synthesis and secretion, Cushing syndrome, pathways in cancer, beta-alanine metabolism, sulfur metabolism, central carbon metabolism in cancer, protein digestion, and absorption pathways.

## 4. Discussion

In this study, the preventive effects of *O*. *sinensis* mycelia on ALI were elaborated in terms of lung coefficients, degree of lung histopathology, and electron microscopic scans. LC-MS was used to detect the changes in metabolites in mouse serum, analyze the differential metabolites and related metabolic pathways in mouse serum, and study the potential mechanism of action of *O*. *sinensis* mycelia for the prevention of ALI. Coronaviruses invade by binding to ACE2, which is abundantly expressed in alveolar type II cells, leading to pathological changes in alveolar epithelial cells, immune hyperactivation, a storm of inflammatory factors, and ultimately, ALI/ARDS [21]. ALI has become a major public health burden worldwide and is characterized by the increased levels of pro-inflammatory transmitters, inflammatory cell infiltration, and alveolar epithelial cell apoptosis [22]. Therefore, controlling abnormal inflammation will effectively improve prognosis [23]. LPS is the main bioactive component of the cell wall of gram-negative bacteria and has long been widely used to induce lung inflammation in mice with ALI owing to its small size, fast growth, and simple source [24]. The H&E staining results of mouse lung tissues showed that the lung tissues of mice in the NS group had normal structures and no obvious pathological changes, but the lung tissues of those in the LPS group showed obvious damage and inflammatory manifestations. A large number of inflammatory cells infiltrated the alveolar cavity, the structural integrity of alveoli was destroyed, and some alveoli collapsed, proving the success of the LPS-induced ALI model in mice.

The above symptoms were significantly reduced in mice in the OS and DXM groups, and the transmission electron microscopy results of lung tissues were consistent with the lung coefficients and H&E staining results, proving that *O*. *sinensis* mycelia could significantly reduce the histopathological changes in the lungs and exert protective effects against ALI. A total of 304 metabolites, which could be regarded as important substances for inducing ALI in mice, were obtained from the LPS and NS groups using multivariate statistical analysis. When LPS stimulates the lungs, the body overproduces reactive oxygen species (ROS), increases malondialdehyde (MDA) and H_2_O_2_ content, and drives the oxidative stress response. The continuously high production of ROS overloads the antioxidant defense system in mice and damages DNA, lipids, and proteins [25]. Large amounts of ROS overload the antioxidant defense system and damage the DNA, lipids, and proteins, thereby driving oxidative stress. Oxidative stress is a critical mechanism in the pathogenesis of ALI. When the production of ROS in the lungs is sustained in large amounts, the lung epithelial and endothelial barrier structures are disrupted, cell membrane permeability is significantly increased, pulmonary edema is exacerbated, and the extent of lung tissue damage is amplified [26]. Therefore, these 304 substances may be related to LPS-induced ALI in mice.

By differential metabolite analysis between the LPS and OS groups, l-histidine, n-isovalerylglycine, indole-3-ethanol, dl-histidine, (hydroxyethyl)met hacrylate, n-acetylvaline, n(6)-[(indol-3-yl)acetyl]-l-lysine, rac-etomidate, cortisol, tetrahydrocortisone, apocynin, 3-hydroxy-3-methylglutaric acid, dimethyl sulfoxide piperonyl sulfoxide, niaprazine, 1-stearoyl-2-arachidonoylplasmenylcholine, gibberellin a24, hydrocortisone cypionate, butylparaben, 7- ketodeoxycholic acid, (-)-nabilone, a-12(13)-epode, and 29 other potential biomarkers were identified. The 10 metabolites with significantly reduced levels in the OS group were mainly amino acid metabolites, including l-histidine, n-isovalerylglycine, dl-histidine, n(6)-[(indol-3-yl)acetyl]-l-lysine, and n-acetylvaline. Among them, l-histidine is a basic amino acid with an imidazole nucleus in the molecule and is a semi-essential amino acid that is necessary for the growth and development of infants and children. It has anti-ulcer effects and is used in the treatment of aging-related diseases, metabolic syndrome, atopic dermatitis, ulcers, inflammatory bowel disease, eye diseases, and neurological disorders [27–29]; it also accelerates erythropoiesis, leukopoiesis, and iron uptake in living organisms. Moreover, l-histidine is a precursor of histamine, myostatin, and other biological substances. It has been suggested that myostatin reduces the formation of MDA, ROS, and glutathione and maintains the activity of glutathione peroxidase, catalase, and SOD, thus alleviating oxidative stress [30]. Research has also confirmed that myostatin can reduce lung injury caused by bleomycin, alleviate the degree of pulmonary edema, and slow pathological changes in the lungs [31]. L-histidine levels were significantly lower in the OS group, suggesting that *O*. *sinensis* mycelia may exert an inhibitory effect on the inflammatory response and oxidative activity by regulating the balance of amino acid-like metabolites. N-isovalerylglycine is an acylglycine that is usually a secondary metabolite of fatty acids and a by-product of the catabolism of the amino acid leucine. As an essential amino acid, leucine not only serves as a substrate for protein synthesis but also stimulates protein synthesis by activating intracellular signal transduction pathways and regulating mRNA translation. Leucine may play a role in repairing ulcerative colonic mucosa by participating in intestinal epithelial cell metabolism and regulating protein turnover [32]. Indole-3-ethanol is an important substance involved in amino acid metabolism and is closely related to tryptophan metabolism [33]. Studies have shown that tryptophan metabolites promote movement and metastasis of cancer cells. For example, in vitro studies have shown that the expression of tryptophan-2,3-dioxygenase in glioblastoma or breast cancer cells promotes tumor cell migration and invasion and overexpression of the rate-limiting enzyme indoleamine-2,3-dioxygenase 1 in lung cancer cells enhances the motility of tumor cells, whereas knockdown reduces their motility [34]. This implies that *O*. *sinensis* mycelia may participate in the prevention of lung injury in mice by regulating amino acid homeostasis and modulating amino acid analogs.

Cortisol is a steroid hormone secreted by adrenocortical fasciculus cells and plays an important role in human substance metabolism and stress responses. Cortisol and tetrahydrocortisone, important components of the steroid hormone biosynthesis pathway, not only exert their corresponding biological effects but also negatively regulate the synthesis and secretion of adrenocorticotropic hormones. Cortisol is important for the treatment of acute lung injury [35]. Cortisol can inhibit the production of oxygen free radicals by normal human peripheral blood polymorphonuclear leukocytes (PMN) for a longer period of time by regulating the activation of NF-κB; cortisol also lessened the extent of lung injury by inhibiting the production of syndromic mediators and overactivation of PMN [36, 37]. The bile acid 7-ketodeoxycholic acid is a steroidal amphiphilic molecule produced by the catabolism of cholesterol. It regulates bile flow and lipid secretion, is essential for the absorption of dietary fats and vitamins, and regulates all key enzymes involved in cholesterol homeostasis. Bile acid receptor agonists have been suggested as a possible treatment for pulmonary inflammatory diseases [38]. The bile acid receptor TGR5 inhibits pulmonary inflammation by inhibiting the activation of the nuclear factor-κB (NF-κB) signaling pathway, while the bile acid receptor farnesol X receptor (FXR) also inhibits pulmonary pro-inflammatory cytokines promoted by lipopolysaccharide by inhibiting cytokine secretion, inhibiting inflammation [39, 40]. Apocynin can inhibit NADPH oxidase activation and increase superoxide dismutase by decreasing the level of total protein and TNF-α; thus, it has a significant protective effect on lipopolysaccharide-induced acute respiratory distress syndrome in mouse models [41], suggesting that the mycelia of *O*. *sinensis* mitigates the inflammatory response and oxidative stress by regulating the levels of apocynin and other substances, which, in turn, have a protective effect on the lungs. These substances exert anti-inflammatory effects by regulating metabolic pathways, including steroid hormone biosynthesis, aldosterone-regulated sodium reabsorption, prostate cancer, cortisol synthesis and secretion, Cushing syndrome, pathways in cancer, beta-alanine metabolism, sulfur metabolism, central carbon metabolism in cancer, and protein digestion and absorption.

In this study, the discovery of potentially active components from *O*. *sinensis* mycelia has important significance in preventing acute lung injury in mice, which will provide support for the clinical application of *O*. *sinensis* mycelia. Our research team will conduct further targeted research on the biological basis of *O*. *sinensis* to prevent acute lung injury based on the analysis of metabolic markers and pathways and their mechanisms of action, providing a reference for the prevention and treatment of ALI.

## Supporting information

S1 Table Information on 1153 identified metabolites. (XLSX)

S2 Table Screening results of differential metabolites between LPS group and NS group. (XLSX)

S3 Table Differential metabolite regulation between LPS and NS groups. (XLSX)

S4 Table Screening results of differential metabolites between LPS group and OS group. (XLSX)

S5 Table Differential metabolite regulation between LPS and OS groups. (XLSX)

S6 Table 10 important pathways enrichment information. (XLSX)
